# Factors affecting the wellbeing of mid-achieving university students: a case study from China

**DOI:** 10.3389/fpsyg.2024.1465209

**Published:** 2024-11-04

**Authors:** Dandan Zou, Zitong Lin, Chen Chen, Huiying Yu

**Affiliations:** ^1^School of Marxism, Guizhou Medical University, Guiyang, China; ^2^College of Literature and Journalism, Qiannan University of Science and Technology, Qiannan, China

**Keywords:** mid-achieving, university students, wellbeing, higher education, psychological health

## Abstract

**Background:**

The study aims to investigate the wellbeing of mid-achieving university students on campus and the factors affecting it. Given that this group represents a large yet often overlooked portion of higher education, the study endeavors to analyze the specific factors affecting their wellbeing to provide insights to foster a more comprehensive and inclusive educational environment.

**Methodology:**

The study adopted a qualitative research method. A total of 30 mid-achieving university students from different departments at Guizhou Medical University in China were interviewed in-depth. The interviews were conducted via the online WeChat platform from 1st March 2024 to 13th September 2024. The semi-structured interviews focused on “moments in campus life that make mid-achieving university students feel happy or joyful.” All interviews were audio-recorded and transcribed into text, which was thoroughly coded and analyzed by the researcher using NVIVO 12 software to comprehensively assess the multiple factors that affect the wellbeing of students.

**Results:**

The wellbeing of mid-achieving university students is affected by a combination of factors, including freedom and autonomy, social interactions, collective activities, campus environment and facilities, and academic achievement.

**Conclusion:**

Enhancing the autonomy of mid-achieving university students can significantly improve their self-efficacy and overall wellbeing. While social interactions and collective activities provide emotional support, they also present interpersonal challenges. Sufficient recreational spaces and a diverse range of food options on campus can help alleviate students’ stress and increase their satisfaction with campus services. Conversely, policies banning motorized bicycles may negatively affect students’ sense of wellbeing. Theoretically, the study contributes to the literature on student wellbeing in higher education psychology, particularly by offering a deeper understanding of the large but often neglected group of mid-achieving students. Practically, the findings emphasize the importance of creating more supportive and responsive educational environments tailored to the needs of these students, thereby facilitating inclusive campus environments and fully harnessing the learning potential of mid-achieving students. The study also proposes specific improvement strategies, such as optimizing campus facilities, enhancing student autonomy, and bolstering social and academic support systems. These measures are expected to directly improve the daily experiences of mid-achieving students and enhance their overall wellbeing.

## 1 Introduction

In higher education, the mental health of university students has always been the focus of educators and researchers alike (McKendrick-Calder and Choate, 2023). A large number of studies have revealed a variety of factors affecting the mental health of these students (Chen et al., 2020). Notably, aspects such as academic stress (Hoover and Lucas, 2023), interpersonal relationships (Marbach and van Zanten, 2023), social activities (Casale et al., 2024), social support networks (Fraser et al., 2022), and career-related expectations and plans have been elucidated as significant contributors to students’ psychological wellbeing (Terrell et al., 2022).

These studies not only provide a nuanced understanding of the prevalent mental health challenges faced by university students but also offer critical insights into their intrinsic needs. However, the rapid evolution of the social landscape and the advent of new technologies have introduced a dynamic array of psychological challenges and pressures faced by university students (Ji et al., 2023; Purnama et al., 2024). Emerging issues such as the impact of digital life (Marjuki et al., 2024), the double-edged sword effect of online socialization (Wong and Liu, 2024), and cultural conflicts arising from globalization (Zalli, 2024) have exerted unprecedented impacts on the mental health of university students.

Therefore, it is essential to revise and enhance research methodologies and theoretical frameworks to better address the mental health disparities among university students from diverse backgrounds and cultural contexts. This evolution in approach will facilitate the development of more precise and personalized support strategies tailored to meet the unique psychological needs of students. By adopting a proactive stance in understanding these multifaceted challenges, we can contribute to creating a more supportive and responsive educational environment that promotes mental wellbeing across the higher education spectrum.

Within the university student community, although there have been a large number of studies focusing on specific groups, such as the psychological conditions of poor students (Kezar et al., 2022; Chin et al., 2024; Tutar et al., 2024), physically disabled students (Daigle et al., 2023), students with special health needs (Koçdar et al., 2024), students from broken families (Widyastuti, 2017), and students with personality disorders (Zhou et al., 2022), there has been a relative lack of research on mid-achieving university students. The factors affecting the wellbeing of this large but often neglected group have not been fully investigated. Mid-achieving students frequently occupy the “mid-zone” between academic performance and societal expectations (Desjardins and Grandbois, 2022; Harpaz et al., 2024) and may present different mental health and wellbeing problems due to unique differences in academic stress, expectation management, and social support systems (Weinstein, 1980). Compared to high-achieving or low-achieving students, they neither belong to the top group nor face serious academic difficulties (Murray and Wren, 2003; Liu et al., 2024a), which distinguishes their unique status as a topic worthy of in-depth exploration in the field of educational psychology.

This study examines the mental health of mid-achieving university students specifically because academic performance within the university student population generally follows a distribution of high, mid, and low achievers (Wong et al., 2024). Notably, the majority of students are situated within the middle range. Despite their ordinary intellectual and academic performance, this does not mean their potential should be ignored. After graduating from university, this group is likely to become the intermediate force of society, which occupies a crucial position in the social structure (Roy, 2012; Warner, 2010; Nowicka, 2024). Therefore, by exploring the factors that affect their wellbeing, we can better understand how to support and guide these future intermediate forces (Lee, 2024) to face future challenges and opportunities with a healthy psychological state and a positive attitude toward life. Therefore, such research not only aims to improve their personal quality of life but can also positively impact the overall harmony and development of society.

The significance of the study lies in (1) developing more targeted teaching strategies and support measures for educators to improve the overall educational experience and satisfaction of university students; (2) providing empirical data support to help educators be more scientific and rational in resource allocation and policy formulation to ensure that mid-achieving students receive resources and support that match their needs and to promote their all-round development; and (3) to further concern and enhance the mental health of intermediate groups, laying the foundation for the healthy growth of intermediate forces in society.

## 2 Literature review

### 2.1 Theory and research status of university students’ wellbeing

The wellbeing of the university student community has become an important topic in educational psychology in wellbeing research (Hernández-Torrano et al., 2020). Wellbeing is usually defined as an individual’s subjective assessment of life quality, covering emotional experience and life satisfaction (Hemarajarajeswari and Kumar Gupta, 2021). In recent years, the study of university students’ wellbeing has gradually gained academic attention, not only because university students are at a critical stage of psychological and physiological development but also because their wellbeing has a profound impact on academic performance and future attitudes toward life and social adaptability (Lyubomirsky, 2001).

It has been shown that university students’ wellbeing is affected by a variety of factors, such as academic stress, social support, economic status (Kellogg, 2021), and individual psychological traits (Hossain et al., 2021). With regard to academic stress, in particular, scholars have found a significant correlation between academic achievement and wellbeing (Shengyao et al., 2024). High-achieving students typically experience higher levels of wellbeing due to their academic performance, whereas academically struggling students are more prone to negative emotions and lower levels of wellbeing due to frequent experiences of failure and stress (Klapp et al., 2023). However, research on the wellbeing of mid-achieving university students in the middle of the academic spectrum remains relatively sparse. Most studies have focused on groups at the extremes of academic performance, resulting in this large mid-achieving group receiving insufficient attention in research.

In addition, theoretical frameworks for university students’ wellbeing usually rely on classical psychological theories, such as Subjective Wellbeing Theory (Proctor, 2024) and Positive Psychology (van Zyl et al., 2023). These theories emphasize that wellbeing is not only a static psychological state but is also affected by dynamic factors such as the learning environment, social networks, and campus climate (Kim et al., 2023). With the ever-changing environment of higher education, it is of great theoretical and practical value to explore the wellbeing of different academic performance groups (e.g., mid-achieving students) and the factors affecting it.

### 2.2 Group characteristics and wellbeing of mid-achieving students

Mid-achieving students, representing a significant subset of the student population across various educational stages, exhibit distinct characteristics and developmental potential (Alipour et al., 2024; Gonder, 1991). Their academic performance falls between outstanding and struggling students, which usually does not attract much attention or receive special support. This group is often categorized as an “invisible group” because they are neither outstanding nor significantly backward in terms of academic performance. Being neither at the top of the class nor showing academic struggles, mid-achieving university students are often marginalized from academic and psychological support systems (Chovan and Freeman, 1993).

It has been shown that mid-achieving university students do not have the high level of self-efficacy that high-achieving students receive, nor do they lack the special counseling and attention that low-achieving students receive when facing academic stress (Shonfeld and Ronen, 2015). In addition, because of their middle-of-the-pack performance, such students may face a unique type of psychological stress in that they have high expectations of themselves, but their academic performance often fails to meet those expectations. This discrepancy between expectations and reality can easily trigger feelings of frustration and anxiety, which in turn can negatively impact their wellbeing (Möller and Pohlmann, 2010; Jin et al., 2007; Bos and Filip, 1984). In addition to academic pressures, mid-achieving students face challenges in social interactions and belonging (Ragusa et al., 2024). Social support plays a critical role in the psychological wellbeing of students. However, mid-achieving students frequently lack adequate support, rendering them more vulnerable to feelings of isolation and low self-esteem (Meskini et al., 2024).

Although there is existing literature on mid-achieving students, research specifically focused on mid-achieving university students remains limited. Despite mid-achieving university students representing a substantial portion of the student population, their needs and problems have failed to receive adequate attention in existing studies (Wintre et al., 2011). According to the existing literature, it suggests that the wellbeing of this group is affected by multiple factors, such as psychological needs and social support. However, the interaction mechanisms among these factors remain insufficiently explored and require further in-depth investigation (Glasserman-Morales et al., 2024).

### 2.3 Factors affecting wellbeing: the perspective of mid-achieving university students

The wellbeing of mid-achieving university students is affected by multiple factors, especially at the academic, social, and psychological levels. First, academic stress is one of the key factors affecting wellbeing. It has been shown that there is a significant correlation between academic achievement and wellbeing, and mid-achieving students are prone to self-doubt and stress because their grades are not up to the top standard. This psychological state may reduce their wellbeing (Plant and Richardson, 1958). These students face stress not only from academics but also from uncertainty about their future development.

Secondly, social support also plays a crucial role in regulating wellbeing. Social support includes not only support from family but also from friends, classmates, and mentors. However, since mid-achieving students do not belong to a particularly prominent group, they may feel neglected or marginalized in their social networks, resulting in their lack of adequate social support, further affecting their psychological wellbeing and life satisfaction (Liu et al., 2024b).

Finally, an individual’s psychological attributes and ways of responding can also affect their wellbeing (Alicke et al., 1995). Research has shown that students with higher levels of psychological resilience are more able to respond positively to academic and life challenges, which in turn maintains higher levels of wellbeing (Albaili, 1997). However, mid-achieving students may lack effective response mechanisms in the face of stress, resulting in a lower sense of wellbeing (Weinstein, 1980; Maroldo, 1986).

In summary, the majority of existing research has focused on the wellbeing of special groups of university students (Mao and Chen, 2023), and there is a lack of research literature on the large group of mid-achieving university students. Despite constituting a significant proportion of the university student population, the factors affecting their wellbeing, psychological needs, and the unique challenges they face lack systematic analysis (Fazio and Palm, 1998; Goldman et al., 1990; BlackDeer et al., 2021). The majority of the existing literature consists of earlier studies, highlighting a gap and limitations in research on mid-achieving university students. The study seeks to extend and update the current research by incorporating novel perspectives and methodologies to deepen the systematic understanding of mid-achieving university students’ wellbeing. The core objective of the study is to refine this field by focusing on the wellbeing of mid-achieving university students and to explore in depth the combined effects of multidimensional factors such as academic stress, social support, and psychological needs on their wellbeing. The case study aims to reveal the complex interactions of these factors, expand the understanding of the psychological status and needs of mid-achieving university students, and provide practical recommendations for educational administrators to help create a more inclusive and supportive educational environment. The innovation of the study lies in its unique focus on the “invisible group.” By integrating psychological theory and educational practice, it explores ways to better support the personal growth and wellbeing of mid-achieving students. This perspective not only provides a theoretical foundation and practical guidance for future educational interventions but also positively contributes to improving the campus life and mental wellbeing of mid-achieving university students.

## 3 Materials and methods

### 3.1 Research design

The study adopts a qualitative research method (Lim, 2024), aiming at exploring and explaining in depth the complex factors affecting the wellbeing of university students. Qualitative research provides insight into a research topic by describing and analyzing specific phenomena in detail, revealing potential problems, and understanding human behavior and mental activity (Theodorou, 2019; Smyth, 2016). Specifically, in the study of subjective and multi-dimensional psychological phenomena such as wellbeing, qualitative methods can reveal the deeper meanings behind the data and help the researcher capture the subtle emotional and cognitive processes, leading to a more comprehensive understanding of the interviewees’ internal experiences. The study selected Chinese university students as the research subjects due to the large size and high diversity of this population (Clemes et al., 2013; Chen et al., 2013). In the Chinese higher education system, which is characterized by intense competition, mid-achieving university students do not receive enough attention, even though they constitute a rather large group (Hu and Wu, 2021). The interviewees in this study were selected from Guizhou Medical University, which offers not only medical programs but also various disciplines in science and the humanities. By choosing students from this comprehensive university, the study is able to reflect a more diverse academic background, ensuring a multidimensional exploration of student wellbeing and a representative analysis. With regard to the particular characteristics of medical students, the study is not limited to the medical field. Instead, interviewees were selected from various disciplines, including medicine, natural sciences, and the humanities, ensuring diversity and broad representativeness in the sample. The selection criteria encompassed not only students with average academic performance but also considered their overall competencies, particularly those demonstrating middle proficiency in areas such as communication, leadership, and innovation. By focusing on this specific group, the study aims to uncover their psychological state and analyze the multiple complex factors affecting their wellbeing.

### 3.2 Data collection procedure

(1) Establishing selection criteria: Defining mid-achieving university students is challenging due to the varying academic competitiveness across different schools, grades, and disciplines. In leading universities, students with mid-achieving grades may still be better in overall performance, whereas in mid-level universities, students with mid-achieving grades may be closer to ordinary students. Therefore, the following selection criteria were established after discussions with three experts in educational psychology and education. First, academic performance was designated as the primary criterion. Specifically, interviewees were required to have academic rankings falling within the middle third to the middle half of their class or cohort (Ho et al., 2019; Slate and Saudargas, 1986). This criterion ensures that interviewees are neither in the high-achieving nor low-achieving groups. Second, the overall quality criterion. In addition to academic achievement, the study will examine the overall ability of the students (Muammar, 2013). The selected mid-achieving students should not exhibit particularly outstanding talent or professional proficiency in any specific area (e.g., music, sports, arts, etc.). Although they may have certain interests, these do not rise to a professional level or attract special attention. Those students with mid-level overall abilities (e.g., communication, leadership, creativity, etc.) can participate in class activities and group discussions. However, they do not take on roles as organizers or core figures. Third, diversity of majors and backgrounds. To ensure the breadth and representativeness of the study sample, interviewees are selected from different disciplines at Guizhou Medical University, including medicine, natural sciences, and the humanities. By selecting interviewees from various academic backgrounds, the study can capture a wider range of perceptions of wellbeing and affecting factors, thus enhancing the study’s representativeness and external validity. (2) Targeting the search: To ensure the formalization and wide dissemination of the information, the study released the recruitment information at Guizhou Medical University in China through the Student Administration Office faculty members. (3) After preliminary communication and selection, a total of 63 students met the selection criteria. With a number of in-depth communications and confirmations, 30 students formally agreed to participate in this interview study. (4) Purpose of the interview: To gain an in-depth understanding of the wellbeing feelings of university students at the mid-achieving level in higher education and their affecting factors, as well as to explore possible strategies for improvement. (5) Interview period: From March 1, 2024, to September 13, 2024. (6) Interview outline: How do you evaluate your current university life? Second, what things have made you the happiest since entering university? Third, please list five moments in campus life that make you happy or joyful and briefly explain why. Fourth, what aspects of campus life could be improved to make you happier and more joyful? Fifth, if you feel unhappy, what factors contribute to that? Please explain. (7) Interview process: By adding students’ personal WeChat accounts, interviewers conducted semistructured interviews using WeChat calls. Each student was interviewed at least two times, with every interview lasting about 2 h. The interviewees’ consent was obtained before each interview, and the interviews were audio-recorded for subsequent analysis.

### 3.3 Data analysis

(1) Data cleaning: The study initially transcribed the audio recordings of the interviews into texts, which were meticulously cleaned to remove irrelevant content, grammatical errors, and any expressions that could lead to misinterpretation. This process ensured the accuracy and relevance of the analyzed data. (2) Code generation: To gain a deeper understanding of the data, the researchers conducted detailed coding of the transcribed texts using NVIVO 12 software. All personal information of the interviewees was anonymized to protect their privacy. The research team identified themes and sub-themes through discussions in online meetings and systematically coded the data. The coding process was completed after all data had been successfully coded to ensure the systematicity and integrity of the study. (3) Theme review: The researcher reviewed and corrected the coding of each theme to verify that it was closely related to the research questions and to ensure that each theme adequately reflected the core meaning of the dataset and maintained the logical coherence of the analysis. (4) Thematic analysis: At this stage, the researcher identified the final themes and sub-themes related to university students’ wellbeing, assigned names to each theme, and drafted detailed analytical reports. These reports explained the logic and significance of the findings, particularly how they relate to the psychological state and wellbeing of the students. (5) Credibility: A rigorous expert validity test was conducted regarding the validity of the interview outline. Three experts in educational psychology and pedagogy reviewed the content of the interview outline, assessed the clarity, relevance, and applicability of the questions, and provided suggestions for improvement. Based on their feedback, the outline was adjusted to ensure that it could effectively capture the key dimensions of students’ wellbeing and enhance the scientific validity and effectiveness of the study. Additionally, to strengthen the credibility of the research further, the following methods were used: first, saturation sampling. The saturation sampling technique was used to continue the interviews until no new information appeared to ensure that the collected materials were comprehensive and in-depth and to enhance the completeness of the research results. The consistency of the data was tested through multiple interviews with the same interviewees by different researchers, with special attention paid to the consistency of the interviewees’ wellbeing before and after. Third, expert review. The study results were presented to three experts in educational psychology and pedagogy, who reviewed the entire data collection, organization, and thematic analysis to ensure the reliability of the research process and conclusions.

## 4 Results

Table 1 demonstrates the sociodemographic characteristics of the 30 survey interviewees (*n* = 30). First, the age distribution of the students was between 19 and 23 years old, which is in line with the typical age range of university students and provides a basis for exploring the impact of different age stages on wellbeing. Second, in terms of the grade level of enrollment, most students were concentrated in the second and third years, and students at this stage usually face the pressure of career choices, which may have a greater impact on their wellbeing. Regarding academic distribution, political science remains the most represented discipline among participants, followed by applied psychology, medical imaging, and biotechnology. The wide range of majors covered, both in the humanities and social sciences, as well as in science, technology, and medicine, indicates that the wellbeing of these students may be related to the diversity of their academic interests and disciplinary backgrounds. Regarding academic performance, the distribution of mid-achieving students is further characterized by the interviewees’ total yearly class size and individual rankings. The majority of students ranked in the middle range of their class or grade, which is consistent with the mid-achieving group that was the focus of the study. These students are not at the top of the academic ladder, nor do they belong to the struggling group, which falls into the “hidden group” that is the main target of this study. By analyzing these characteristics, the study not only explores in depth the performance and potential effects on the wellbeing of mid-achieving university students but also provides a solid foundation for subsequent analyses.

**Table 1:** Sociodemographic characteristics of the qualitative study participants (*n* = 30).

Code	Gender	Age	University	University year	Major	School	Total enrollment in the year	Ranking
A1	F	22	Guizhou Medical University	Year 2	Political Science	School of Marxism	62	33
A2	F	21	Guizhou Medical University	Year 2	Political Science	School of Marxism	62	34
A3	F	21	Guizhou Medical University	Year 2	Political Science	School of Marxism	62	21
A4	F	21	Guizhou Medical University	Year 2	Political Science	School of Marxism	62	23
A5	F	20	Guizhou Medical University	Year 2	Political Science	School of Marxism	62	36
A6	F	20	Guizhou Medical University	Year 2	Political Science	School of Marxism	62	35
A7	F	21	Guizhou Medical University	Year 2	Political Science	School of Marxism	62	24
A8	F	22	Guizhou Medical University	Year 2	Political Science	School of Marxism	62	36
A9	F	21	Guizhou Medical University	Year 2	Political Science	School of Marxism	62	27
A10	M	19	Guizhou Medical University	Year 1	Political Science	School of Marxism	59	26
A11	F	19	Guizhou Medical University	Year 1	Political Science	School of Marxism	59	21
A12	M	23	Guizhou Medical University	Year 3	Political Science	School of Marxism	99	50
A13	F	23	Guizhou Medical University	Year 3	Political Science	School of Marxism	99	33
A14	M	21	Guizhou Medical University	Year 2	Medical Imaging	School of Medical Imaging	100	50
A15	F	23	Guizhou Medical University	Year 3	Applied Psychology	School of Medical Humanities	66	38
A16	F	23	Guizhou Medical University	Year 3	Applied Psychology	School of Medical Humanities	66	34
A17	M	21	Guizhou Medical University	Year 2	Biomedical Engineering	School of Bioengineering	89	43
A18	F	20	Guizhou Medical University	Year 2	Biotechnology	School of Oceanography	129	59
A19	F	20	Guizhou Medical University	Year 2	Biotechnology	School of Oceanography	129	77
A20	M	20	Guizhou Medical University	Year 2	Biotechnology	School of Oceanography	129	42
A21	M	21	Guizhou Medical University	Year 2	Biotechnology	School of Oceanography	129	89
A22	M	21	Guizhou Medical University	Year 2	Biotechnology	School of Oceanography	129	76
A23	M	21	Guizhou Medical University	Year 2	Biotechnology	School of Oceanography	129	51
A24	M	21	Guizhou Medical University	Year 2	Biotechnology	School of Oceanography	129	93
A25	F	21	Guizhou Medical University	Year 2	Pharmacy	School of Ethnic Medicine	80	37
A26	M	21	Guizhou Medical University	Year 2	French	School of Foreign Languages	70	48
A27	F	21	Guizhou Medical University	Year 2	Logistics Management	School of Management	120	96
A28	F	21	Guizhou Medical University	Year 2	Nursing	School of Nursing	803	624
A29	F	21	Guizhou Medical University	Year 2	English	School of Foreign Languages	80	18
A30	M	21	Guizhou Medical University	Year 2	Police Command and Tactics	Department of Police Skills Training	650	291

### 4.1 Freedom and autonomy

In higher education, freedom and autonomy significantly impact the wellbeing of mid-achieving university students. Unlike the rigidity and standardization of high school education, university education offers greater personal freedom and autonomy, which are essential to students’ personal growth and wellbeing. China’s university entrance examination system, known for its intense competition and high pressure, typically requires high school students to study in a highly structured academic environment, where personal interests and choices are frequently sacrificed in pursuit of higher grades. Nevertheless, this situation has changed dramatically at the university. Not only do students have the ability to choose a major that interests them, but they also have more flexibility in their course choices and learning styles, and this enhanced freedom allows for personal exploration and goal-setting based on their interests and career goals (Chen et al., 2020). The new freedoms and autonomy universities offer are key to their personal growth and pursuit of wellbeing.

#### 4.1.1 Increased freedom

Compared to high school life, university life offers a higher level of freedom where students can organize their time and activities according to their interests.

*“I think university life is great. I can do what I want and like every day instead of being constrained like in high school. It's very relaxing. This is really the life I imagined. There is no one to force me to do anything. I can do what I am interested in and work towards what I am interested in. It's really a good life.” (A5)*

*“I think my university life is relatively free and happy. I can do what I want to do and what's interesting in addition to my studies and gradually clarify my goals and directions in the process of exploration. It's a great feeling, and although it can be tiring a lot of the time, I think it's a necessary part of my growth.” (A1)*

*“Feeling fine with my life now, it is the type of life I used to want a lot, that is freedom, no one constrains me, which is the very comfortable status of life.” (A17)*

#### 4.1.2 Personal exploration and goal setting

In university, students discover and pursue their interests, which not only strengthens their sense of self-fulfillment but also assists them in formulating and achieving personal goals.

*“I have the freedom to engage in club activities that interest me, which has helped me to gradually clarify my future goals and direction, and I can now move towards that goal with one foot in front of the other.” (A3)*

*"My university life is very fulfilling, and I have the chance to explore various new disciplines and knowledge. It is incredibly important to pursue one's interests through free exploration because interest can be the best teacher, providing true satisfaction in the process of pursuit." (A11)*

#### 4.1.3 The importance of autonomy in decision-making

The autonomy of being able to choose the activities or fields of study in which they participate makes their academic and social activities more meaningful and satisfying.

*“I think my university life is looking relatively good so far. I can find time to read books after class every day. Also, I can save up some pocket money every month to take a trip.” (A9)*

*“I think university life is quite peaceful at the moment; there is no pressure, so it's easier to get through. Clubs, activities, competitions, and whatever else in my free time are ways to spend my time.” (A10)*

### 4.2 Social interactions and collective activities

In higher education, social interactions and collective activities enhance the wellbeing of mid-achieving university students, emphasizing overall development rather than being limited to academic achievement. Friends and social support are key to adapting to new environments and overcoming difficulties in university life, helping students balance academics and life (Kaufman and Nemeroff, 2024). Participation in clubs and team activities not only develops teamwork and leadership skills but also enhances students’ sense of belonging and pride. Some students release stress by participating in e-sports games, such as League of Legends, which are highly cooperative. Through these games, they build and maintain strong interpersonal relationships.

#### 4.2.1 Friends and social support

Creating a supportive social network, spending time with friends, and sharing moods are important for students’ emotional and mental health.

*“I think I am the happiest when I can make like-minded friends. I travel and improve with my new friends. They tell me stories that I've never heard before, and we share food and funny stories from each other's hometowns. I'm no longer on my own, but I'm surrounded by so many friends who are just like me and fight with me, and it's a moment that makes me so happy.” (A2)*

*“I think when it rains, I guess, I can chat with everyone inside the dormitory and share my feelings of joy and anxiety. Lying on the bed, listening to the rain, and slowly falling asleep, that is such a relaxing thing.” (A6)*

*“Making friends with more great people, I can learn what kind of mindset they have.” (A13)*

*“The happiest thing is that I met a lot of friends who have the same hobbies, and I won't be lonely in the future.” (A21)*

*“Meeting new friends eases that sadness of leaving former good friends.” (A25)*

#### 4.2.2 Collective activities and team honor

Participating in clubs and collective activities, such as sports games and competitions, not only promotes teamwork but also brings individuals a sense of achievement and honor.

*“During the summer, I participated in the 'Supporting Villages' social practice, which brings us into contact with different people and things, and the experience of helping others is very precious.” (A29)*

*"Participating in school and university activities, working together with classmates and friends toward a common goal is truly great. I really enjoy the moments of receiving recognition, whether it is for the group or individually, as both are meaningful experiences for me."(A3)*

*"The first time I participated in the Three Rural Support Project and Volunteer Teaching Program was the happiest time for me. I really enjoy and actively participate in these activities. I feel that I gain a lot from them, things that I cannot learn from books, and I also get to meet many people through team interactions." (A12)*

#### 4.2.3 Interpersonal relationship support

Students need to manage and maintain interpersonal relationships, as positive relationships can be a source of wellbeing, whereas relational issues may contribute to stress and dissatisfaction.

*"In terms of relationships, I enjoy making friends, but it has also created many issues. My friends might have conflicts, and I find myself stuck in the middle. I have to spend a lot of time mediating between them, which disrupts my study and rest time." (A2)*

*"Handling relationships is really difficult for me. I can't manage to cater to everyone's feelings, and some of them may hurt me with harsh words because of that." (A4)*

*"My happiest moments are probably after finishing a day of studying and work, lying in the dorm and playing League of Legends." (A19)*

*"I really enjoy the atmosphere of playing games together in the dorm at night." (A24)*

*“Carrying the whole game, putting together a winning streak, and everyone being willing to play the game with me was very satisfying.” (A20)*

*"I can't stand my annoying roommate. It makes me not want to stay in the dorm at all." (A26)*

### 4.3 Campus environment and facilities

In higher education, the campus environment and facilities profoundly impact the wellbeing of mid-achieving university students. A comfortable campus environment, such as high-quality cafeteria food and good public facilities, directly increases students’ daily life satisfaction. The campus’s natural environment, including the degree of greenery and walking routes, provides students with space for relaxation and pleasure, helping them decompress and regulate their psychological status by taking a walk after classes or meals. The rainy weather relieves students’ stress and makes it easier to fall asleep and relax. However, improvements in campus transportation convenience and infrastructure, such as more efficient courier services and accessible public transportation, are necessary to enhance students’ overall wellbeing. These factors collectively form a supportive learning and living environment that promotes students’ mental health and academic success.

#### 4.3.1 Impact of campus facilities

A comfortable campus environment, such as the quality of food in the cafeteria and public facilities, directly impacts students’ daily life satisfaction.

*"When I eat delicious food in the cafeteria, I feel like good food can heal everything. It helps me temporarily disconnect from heavy coursework, allowing me to relax and feel less stressed." (A1)*

*"Having good food in the cafeteria lifts my mood. When I'm in a good mood, I feel more motivated to do everything." (A15)*

*"They should build more pathways because the campus is too big. Walking along the main roads is tiring, and having more shortcuts would make it more convenient. Also, adding more benches would be great, so when you're tired from walking, you can sit and rest, making the walk less rushed." (A6)*

#### 4.3.2 Importance of the natural environment

The natural environment on campus, such as walking routes and greenery, provides spaces for relaxation and enjoyment, which contribute to students’ wellbeing.

*"Blowing the evening breeze with friends on the campus lawn makes all my worries disappear. I think the school should introduce more plants to improve the environment. A beautiful environment in our daily school life benefits our mental and physical health." (A14)*

*"Walking by the lake after dinner, feeling the breeze, helps me forget my troubles and relax. Building pavilions on campus would provide shade and give us better opportunities to enjoy the scenery." (A9)*

*"Taking a walk in the evening, feeling the breeze, walking slowly—it's very pleasant." (A7)*

*"Walking on the track at night, feeling the breeze, and daydreaming about the future, even though I might not achieve it, the fantasy always lifts my spirits." (A15)*

#### 4.3.3 Impact of weather

Rainy days offer students a sense of coziness and relaxation, with the sound of rain serving as natural white noise that aids in relaxation and rest. Moreover, rainy weather fosters emotional connections during interactions with roommates. However, puddles and muddy pathways can cause inconvenience and dirty clothing, negatively impacting students’ moods.

*"I feel very happy when it's raining, and I'm in the dorm watching movies and snacking. The laughter and casual chats with my roommates make me feel like I'm not alone and that I have a trustworthy group of friends with me. At that moment, I don’t have to worry about anything. I just enjoy the time." (A3)*

*"On rainy days, I can chat with everyone in the dorm, sharing my joys and anxieties. Then I lie in bed, listening to the white noise of the rain as I slowly fall asleep. It’s very relaxing." (A6)*

*"When it rains, I like listening to the rain while having a drink in the dorm. It’s a really nice feeling." (A7)*

*"On rainy days, I can lie down and listen to the sound of the rain outside. The natural white noise is very soothing." (A17)*

*"I think the school’s landscaping needs improvement. Otherwise, when it rains or during extreme weather, there’s a lot of water and mud on the roads, which not only affects aesthetics but also makes it inconvenient to walk." (A12)*

#### 4.3.4 Transportation and infrastructure

The convenience of campus transportation and the adequacy of infrastructure, such as courier services and public transportation, directly impact students’ daily lives and overall satisfaction.

*"All couriers should be allowed to enter the school. The locations of the other two couriers are too far from me, which is very inconvenient. It would be great if the remaining couriers could enter the campus pickup station." (A7)*

*"Transportation within the school is very inconvenient. There are too few campus shuttles, and the waiting lines are long. Plus, with the ban on motorcycles and electric scooters, it's even more inconvenient. The campus is huge, and if I don’t take a shuttle, it’s extremely inconvenient to get anywhere, and it wastes a lot of time." (A4)*

*"The campus is too far from the city, and transportation is very inconvenient. On top of that, the recent ban on motorcycles and electric scooters has made it difficult to get around campus, let alone go off-campus." (A8)*

*"I find the transportation quite inconvenient. The campus is large, but there are few campus shuttles, and they mostly follow a single route, which isn't practical. Off-campus transportation is also difficult—waiting for the bus outside the school is time-consuming, and taking unlicensed taxis is unsafe and expensive." (A12)*

### 4.4 Academics and achievement

When there are no early morning classes, students are able to sleep until they wake up naturally. Sleeping adequately makes them feel comfortable and energized, which significantly improves their sense of wellbeing. Moreover, academic success, such as passing important exams or completing courses, brings a strong sense of satisfaction and pride, which positively impacts their self-confidence and mental health. However, as graduation and the job market approach, students face fierce employment competition, leading to increased academic pressure and common feelings of anxiety, especially around final exams. In university life, support and recognition from teachers are also crucial. Positive feedback and validation from instructors can significantly boost students’ satisfaction and confidence, while a lack of support may lead to feelings of frustration and insecurity. Therefore, academic achievements and challenges, along with teacher support, play a key role in shaping students’ learning experiences and overall wellbeing.

#### 4.4.1 No early morning classes

When there are no early morning classes, students can sleep in, which makes them feel very comfortable. Adequate sleep not only energizes them but also significantly enhances their sense of wellbeing.

*"I'm really happy when there are fewer classes. Not having an 8 AM class is such a relief." (A4)*

*"No early classes mean I can wake up naturally." (A25)*

*"Waking up naturally in the dorm and feeling energized is really nice. If I don’t sleep well, I feel tired, and I’m in no mood to feel happy." (A9)*

*“Being able to sleep until naturally awake on weekends dissipates all the tiredness of the week.” (A15)*

#### 4.4.2 Academic achievement

Successfully completing courses and exams, especially major academic milestones such as passing the CET-4 (University English Test Band 4), brings students a strong sense of satisfaction and pride.

*"After achieving good results through my own efforts, I feel very fulfilled because it’s a recognition of my hard work. For instance, when I received the National Inspirational Scholarship for the first time, I felt like my effort was acknowledged and rewarded. That feeling of being recognized and praised is really great." (A12)*

*"There are many things that make me happy, and getting good grades is definitely one of them. It feels rewarding to see the results of my hard work, and I feel like my efforts weren’t in vain." (A16)*

*"What makes me happiest is having the freedom to manage my free time. Unlike the restricted life in high school, at university, I can arrange my time based on my needs and preferences." (A18)*

*"Everything brings joy, but if I had to pick the ‘most’ joyful moment, it would probably be the sense of breaking through my limits every time I complete an experiment." (A22)*

"Winning in those competitions also indirectly proved my abilities and skills, and it became something I could proudly talk about." (A23)

#### 4.4.3 Managing academic pressure

While academic achievement is a source of wellbeing for students, excessive academic pressure and exam anxiety are common obstacles to their wellbeing.

*"High academic pressure makes me feel very uncomfortable. The anxiety it causes even leads to physical discomfort, especially during finals. I often feel so anxious that I can’t rest well, and sometimes I barely eat, leaving me in a bad mood." (A2)*

"The coursework is too overwhelming. I feel like the pressure it creates is something that can’t be relieved in a short period of time, especially during the review week before exams. It feels like ‘torture’ to me. I can't exercise, and the mental stress keeps building, making me very anxious." (A5)

*"The academic pressure is quite high. With so many assignments, I easily get exhausted." (A14)*

*"Not failing the exams means I don’t have to worry about studying for retakes during the break." (A27)*

#### 4.4.4 Teacher support and recognition

Receiving praise and recognition from teachers can significantly boost students’ confidence and satisfaction. Conversely, a lack of support and acknowledgment may lead to feelings of frustration.

*"Being praised by the teacher in class made me feel like my efforts and ideas were recognized, and that made me really happy." (A30)*

*"Getting recognition and praise in both academic work and activities makes me feel valued, which provides important psychological support." (A28)*

## 5 Discussion

This study reveals that the desire for autonomy among mid-achieving university students is evident not only in their pursuit of academic freedom but also in their control over personal life choices. This desire is particularly important in the growth and development process of university students because it is directly related to their self-concept, career planning, and personal growth (Ito and Kodama, 2006). First, a heightened sense of autonomy is a notable change for mid-achieving university students in their transition to university life. High school students typically face a rigorous schedule and norms of life, whereas university provides a relatively flexible environment that allows students to make independent decisions based on their personal interests and needs, offering a broader selection of courses, interest groups, and social activities. Second, mid-achieving university students show a strong desire for freedom, which not only manifests itself in freedom of academic choice but also broadly affects their lifestyles, time management, and social activities. This freedom allows students to explore their personal interests and potentials in-depth and engage in more personalized self-development. For example, by joining different clubs and organizations or participating in volunteer activities, students not only enrich their university experience but also make progress in building social networks and developing teamwork skills (Botha et al., 2019). Finally, the positive effect of having autonomy is reflected in the ability of students to make decisions in a broader range of areas that are consistent with their interests and career plans. This empowered choice directly enhances students’ initiative and motivation in their academic and personal development. The expansion of autonomous choices not only increases students’ satisfaction with their academics and personal lives but also improves their ability to solve problems and meet future challenges. As a result, students’ self-efficacy is significantly strengthened, ultimately leading to increased wellbeing and quality of life.

The wellbeing of mid-achieving university students is impacted by collective activities and social interactions that not only provide realistic opportunities for learning and growth but also bring social complexities and challenges. Through participation in collective activities such as clubs, project work, or volunteer service, students provide the opportunity to develop skills in collaboration, leadership, and problem-solving. These are valuable experiences that are difficult to acquire within the confines of the classroom. The sense of participation and practical experience strengthens students’ social integration and self-efficacy, enabling them to recognize their contributions to society more effectively. This, in turn, enhances their personal satisfaction and overall wellbeing. Mid-achieving university students may encounter interpersonal conflicts and tensions as they expand their social circles and increase their social activities (Myburgh et al., 2019), sometimes negatively impacting their emotional health and academic performance. For example, excess social demands may distract students from their studies, whereas interpersonal conflicts or adverse social experiences may lead to anxiety and frustration. Therefore, despite the significant role that collective activities and social interactions play in promoting the personal growth and professional competence development of mid-achieving university students, they are also double-edged swords that have the potential to either increase students’ sense of wellbeing or be a source of emotional and psychological stress for them.

Mid-achieving university students remarkably valued the campus environment, where the scenery of the walking routes and the availability of high-quality food were cited as effective stress relievers. The majority of students interviewed indicated that being able to enjoy quiet walks and good food on campus greatly enhanced the quality of their daily lives and their mental health. This advantage of the campus environment not only provided students with space to relax but also became an important factor in their evaluation of their school-life experience (McDonald-Yale and Birchall, 2021). However, the accessibility of the campus and certain policies, especially regulations restricting electric vehicles, pose a challenge to students’ wellbeing. These policies constrain students’ freedom of movement, increasing the challenges associated with mobility on and off campus, consequently impacting their overall satisfaction. Therefore, although certain environmental factors on campus, such as walking routes and cafeteria quality, contribute positively to students’ experiences, inconvenient transportation conditions and strict management policies may diminish these benefits. This suggests that administrators should balance convenience and safety when formulating policies to improve students’ overall experience of living on campus (Byrd and McKinney, 2012; Lin and Lin, 2022).

Though mid-achieving university students emphasize diverse experiences in their campus life, grades and academic achievement also occupy the core of their concerns. This group of students faces considerable academic pressure as maintaining or improving grades becomes an important part of their daily lives in a competitive academic environment. This pressure stems from concerns about future career prospects and expectations from peers and teachers. Although striving to improve academic performance is supposed to be a positive behavior, constant pressure can sometimes lead to anxiety and stress overload, which can affect students’ mental health and overall wellbeing (Wang, 2022; Shi and Fang, 2024). Therefore, for these students, learning how to manage and cope with academic stress and balance their academic and personal lives is not only part of their daily challenges but also a key factor in their long-term wellbeing and quality of life. Universities and educators should offer targeted support and resources, such as psychological counseling services and skills workshops, to assist students in effectively managing these pressures. By doing so, they can enhance students’ academic experiences and improve their overall life satisfaction.

The study provides some new findings and refines previous research on the wellbeing of mid-achieving university students. First, the study found that mid-achieving university students strongly desire freedom and autonomy. This contrasts sharply with the constraints and pressures experienced during high school, as the greater autonomy in university life allows students to manage their time independently, thereby substantially improving their wellbeing. Moreover, this finding challenges previous research that primarily focused on the impact of academic pressure and achievement on student wellbeing, highlighting the importance of autonomy and freedom of choice for mental health. Second, social interactions and gaming activities were found to be important means of emotion regulation for mid-achieving students. A proportion of students released stress by playing competitive games (e.g., League of Legends) while fostering and maintaining interpersonal relationships through these games. This is a departure from the traditional view that academics and socialization are separate, illustrating the unique role of gaming as a socialization tool.

Additionally, this study found that mid-achieving university students valued the campus environment and facilities, particularly campus walking routes, greenery, and climate, as having a direct impact on their wellbeing. It has become a new finding that rainy days bring a sense of relaxation, and the sound of rain is regarded as a natural white noise that helps students relax during a stressful academic life. However, infrastructural problems in inclement weather, such as stagnant water and muddy roads, can be inconvenient and affect their mood. Adequate sleep is also a key factor, with students longing for more time to rest, reflecting the distress caused by intense courses and employment pressures. Delicious cafeteria meals, though seemingly insignificant, were also cited by students as an important factor in enhancing their sense of wellbeing. This suggests that the campus environment, which is not the most important determinant of wellbeing, affects students’ psychological state and emotional experience. These findings provide new perspectives on the management and services of universities, suggesting that improving campus environments, facilities, and living conditions can effectively upgrade the overall wellbeing of mid-achieving university students.

The study presents significant findings and recommendations regarding theoretical contributions and practical implications. Regarding autonomy and freedom, the research indicates that enhancing freedom and decision-making autonomy plays a crucial role in improving the wellbeing of mid-achieving university students. The findings extend theoretical knowledge about individual autonomy in wellbeing and emphasize the importance of autonomous choice for students’ psychological wellbeing and self-actualization. Social interaction and collective activities were also shown to be important factors in enhancing wellbeing, especially with the support of friends and teams, and collective activity promotes the development of interpersonal relationships and reinforces a sense of team honor, suggesting that social support is not only helpful for high-achieving students but also critical for mid-achieving students. The impact of the campus environment and facilities has also been prominently highlighted, further contributing to the theoretical understanding of how the physical environment affects students’ psychological wellbeing. The findings indicate that improvements in natural surroundings, climate conditions, and campus infrastructure can significantly enhance students’ overall sense of wellbeing. Finally, academic achievement plays a dual role in affecting wellbeing: a sense of academic accomplishment can enhance wellbeing, whereas academic pressure detracts from it. The findings underscore the crucial moderating role of teacher support and recognition in this process, which should not be overlooked. In terms of practical implications, this study provides several suggestions for improvement for university administrators. First, universities should give students more autonomy to make their own decisions in course scheduling, especially considering the rationality of course scheduling and allowing students to have enough sleep to heighten their sense of autonomy and wellbeing (Liu et al., 2022; Cedillo et al., 2024). Second, more social interactions and collective activities should be encouraged to promote students’ social support networks and strengthen their sense of belonging and team honor. Improvements in campus facilities and the natural environment are also crucial, especially regarding climatic conditions and infrastructure, which can significantly improve students’ daily life experiences. Finally, schools should also strengthen psychological support and academic counseling for mid-achieving students to help them better manage academic stress and promote a sense of academic achievement, thereby enhancing overall wellbeing. Universities can create a more supportive environment for mid-achieving students through these improvements.

The study has the following limitations: first, sample limitations. The sample of this study focuses on a group of mid-achieving university students and fails to cover students with higher or lower grades. Therefore, the study’s results may not fully reflect the wellbeing status of all university students. Future studies should expand the sample to cover students with different academic performances and diverse backgrounds to ensure the broad applicability and comprehensiveness of the findings. In addition, the small sample size (*n* = 30) may limit the generalizability of the results, and the robustness of the results can be elevated by increasing the sample size in the future. Second, the data collection method has limitations. This study relied heavily on self-report data, and although this method is commonly used in qualitative research, self-reports may be influenced by social desirability effects and participants’ self-perceptions, resulting in biased data. Students may adjust their responses to align with social expectations or subjectively assess their emotional states, which could potentially affect the accuracy and objectivity of the data. Future research could incorporate objective data sources such as behavioral observations, psychological assessments, or physiological data to provide a more comprehensive analysis. Third, the limitations of the interview method. Conducting interviews using the WeChat platform is an innovative and practical approach in the Chinese context, especially given the geographic location and time constraints. However, the informality of the WeChat platform may affect the depth of responses, and participants may tend to simplify or shorten their responses in an informal setting, reducing the richness of the data. Therefore, future research should consider combining multiple data collection methods, such as face-to-face or more structured online interviews, to increase the depth and detail of the data. Additionally, the environment of the WeChat platform interviews may have affected participant focus and response quality, which could be improved by conducting the interviews in a controlled environment.

## 6 Conclusion

This study found that the wellbeing of mid-achieving university students is affected by a variety of factors, including freedom and autonomy, social interaction, campus environment, and academic achievement. Based on these findings, the study presents significant practical implications. First, enhancing autonomy within the university environment allows students greater freedom in selecting and organizing their academic and personal lives, thereby increasing their sense of self-efficacy and overall wellbeing. Therefore, it is recommended that universities provide students with greater autonomy in course selection and activity scheduling, reduce unnecessary restrictions, and implement course arrangements that ensure students have adequate time for rest and sleep. Second, collective activities and social interactions not only provide students with opportunities for practice and growth but also support them emotionally. Therefore, universities should further strengthen the organization of collective activities and create more social opportunities to help students build positive interpersonal networks. The quality of the campus environment significantly impacts students’ wellbeing, especially factors such as walking routes, campus greenery, and cafeteria food. Therefore, it is recommended that universities continue to optimize campus infrastructure and living conditions to improve students’ daily life experiences. Negative factors, such as transportation accessibility and policy constraints, emphasize the need for universities to carefully consider students’ practical needs when developing management policies to minimize potential negative impacts on their quality of life. Finally, although academic stress is a significant impediment to student wellbeing, by providing personalized academic guidance, enhancing psychological support systems, and optimizing learning resources, educators can help students alleviate stress and enhance their overall wellbeing. By implementing these improvements, university administrators can create a more supportive learning and living environment for mid-achieving university students, thereby enhancing their wellbeing and overall development.

## Data Availability

The original contributions presented in the study are included in the article/supplementary material. Further inquiries can be directed to the corresponding author.
